# mTOR-Mediated Antioxidant Activation in Solid Tumor Radioresistance

**DOI:** 10.1155/2019/5956867

**Published:** 2019-12-20

**Authors:** Yunseo Woo, Hyo-Ji Lee, Young Mee Jung, Yu-Jin Jung

**Affiliations:** ^1^Department of Biological Sciences and Institute of Life Science, Kangwon National University, Chuncheon 24341, Republic of Korea; ^2^Department of Chemistry, Kangwon National University, Chuncheon 24341, Republic of Korea

## Abstract

Radiotherapy is widely used for the treatment of cancer patients, but tumor radioresistance presents serious therapy challenges. Tumor radioresistance is closely related to high levels of mTOR signaling in tumor tissues. Therefore, targeting the mTOR pathway might be a strategy to promote solid tumor sensitivity to ionizing radiation. Radioresistance is associated with enhanced antioxidant mechanisms in cancer cells. Therefore, examination of the relationship between mTOR signaling and antioxidant mechanism-linked radioresistance is required for effective radiotherapy. In particular, the effect of mTOR signaling on antioxidant glutathione induction by the Keap1-NRF2-xCT pathway is described in this review. This review is expected to assist in the identification of therapeutic adjuvants to increase the efficacy of radiotherapy.

## 1. Introduction

Ionizing radiation is a therapeutic method that can induce cell death in tumors through direct or indirect damage, such as covalent bond breakage and reactive oxygen species (ROS) accumulation in cells [1–3]. Although radiotherapy efficacy is somewhat different depending on tumor type, excellent outcomes have been shown in many clinical reports [4]. Therefore, approximately 50% of patients with solid tumors undergo radiotherapy [5]. However, radioresistance has been observed in several types of tumors, including melanoma [6, 7]. In general, radiation upregulates radioresistant genes in tumor cells [8]. These upregulated genes are closely related to survival mechanisms, such as DNA repair, metabolic changes, tumor recurrence, and malignant transformation [9]. Thus, controlling the signals associated with radiation-induced resistant genes might be a major strategy in radiotherapy to reduce the irradiation burden in the host [10, 11]. For this reason, the discovery of adjuvants targeting radioresistant molecules is important for effective radiotherapy [12, 13].

**Figure 1:**
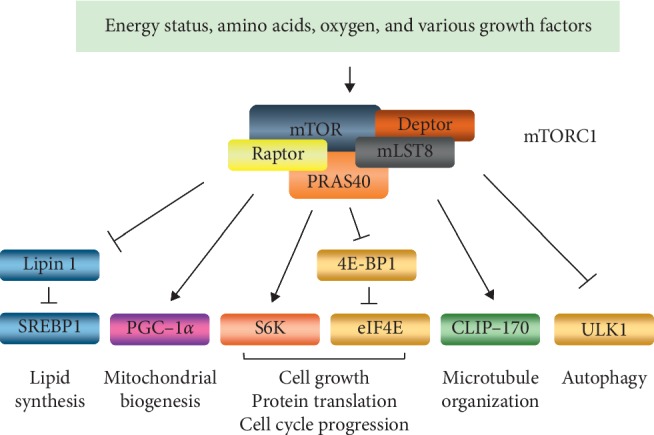
The functions of mTOR complex 1 (mTORC1). Intrinsic or extrinsic stimulators, such as energy levels, amino acids, oxygen levels, and various growth factors, induce mTORC1 activation. Once mTORC1 signaling is activated, lipid synthesis, mitochondrial biogenesis, and protein synthesis are promoted in the cells. mTORC1 also organizes microtubules and suppresses autophagy.

mTOR is a major signaling protein that can affect cell survival through interaction with other cellular signal cascades. mTOR, which is a serine-threonine kinase, can form two distinct multiprotein complexes, mTOR complex 1 (mTORC1) and mTOR complex 2 (mTORC2) [14]. mTORC1 consists of five proteins, namely, mammalian target of rapamycin (mTOR), regulatory-associated protein of mTOR (Raptor), proline-rich AKT substrate 40 kDa (PRAS40), DEP-domain-containing mTOR-interacting protein (Deptor), and mammalian lethal with Sec13 protein 8 (mLST8) [15]. In general, mTORC1 activation is known to be regulated by energy status, amino acids, oxygen conditions, and various growth factors (Figure 1) [16]. Once mTORC1 is activated, lipid synthesis, protein synthesis, mitochondrial metabolism, and microtubule organization can be induced in cells [17]. However, autophagy is actually inhibited by mTORC1 as indicated in Figure 1. Sterol regulatory element-binding protein 1 (SREBP1) can stimulate lipid synthesis, but it is suppressed by lipin 1 in the nucleus [18]. However, activated mTORC1 promotes SREBP1-mediated lipid synthesis through inhibiting lipin 1 translocation into the nucleus by inducing its phosphorylation [19]. Peroxisome proliferator-activated receptor-*γ* coactivator-1*α* (PGC-1*α*) controls mitochondrial biogenesis and promotes mitochondrial metabolism [20]. In addition, PGC-1*α* regulates ROS generated during mitochondrial oxidative phosphorylation (OXPHOS) [21]. mTORC1 activates these functions of PGC-1*α* [22]. Furthermore, mTORC1 not only promotes microtubule organization through cytoplasmic linker protein-170 (CLIP-170) phosphorylation but also controls protein synthesis via the ribosomal protein S6 kinase beta-1 (S6K1) and the eukaryotic translation initiation factor 4E (eIF4E)-binding protein 1 (4E-BP1) [23, 24]. Moreover, mTORC1 inhibits Unc-51-like autophagy activating kinase 1- (ULK1-) mediated autophagy [25]. Because mTOR is connected to networks formed by various signaling pathways in the cell survival process, mTOR signaling is important in tumor studies.

The PI3K/AKT/mTOR pathway is associated with tumorigenesis, metastasis, and tumor therapeutic resistance [26]. In particular, the survival of tumor patients is affected by upregulated PI3K/AKT/mTOR cascade activity. mTOR phosphorylation was observed in 64.1% of tumor tissues obtained from gallbladder cancer (GBC) patients, and mTOR phosphorylation levels were associated with poor prognosis in these patients [27]. The poor survival of human hepatocellular carcinoma (HCC) patients was related to enhanced levels of phospho-AKT (491/528; 92.99%) and phospho-S6 (466/528; 88.26%) in their tumor tissues [28]. The induction of mTOR activation was also associated with poor prognosis in solid tumor patients, such as breast cancer, melanoma, gastric cancer, and urothelial carcinoma [29–32]. Although enhanced mTOR signaling activation has been reported to not be associated with the survival of some tumor patients, targeting mTOR signaling is still a reasonable approach for tumor radiotherapy [33–36]. Any protein in the PI3K/AKT/mTOR pathway can be targeted for the clinical inhibition of mTOR activation in tumor tissues [37, 38]. In particular, clinically targeting mTOR signaling during radiotherapy could increase tumor radiosensitivity [39]. Sirolimus analogs (rapalogs and mTOR inhibitors), such as everolimus, temsirolimus, and ridaforolimus, not only improved radiotherapy efficiency but also reduced tumor recurrence [40–43]. These previous studies implicate that blocking mTOR signaling in tumors might be an effective strategy in radiotherapy.

High levels of antioxidant molecules in tumors have been identified as factors that make antitumor therapy difficult in many previous studies [44, 45]. Therefore, targeting antioxidant mechanisms might be a good therapeutic strategy for efficient radiotherapy [46]. Recent studies have reported that antioxidant mechanisms can be regulated by mTOR signaling [47–49]. In this review, the effects of mTOR inhibition on the antioxidative Keap1-NRF2 pathway in solid tumors during radiotherapy and the underlying mechanisms are assessed.

## 2. mTOR Activates Antioxidant Defense Mechanisms in Radioresistant Solid Tumors

### 2.1. Mitochondrial ROS Generation and the KEAP1-NRF2 Pathway

**Figure 2:**
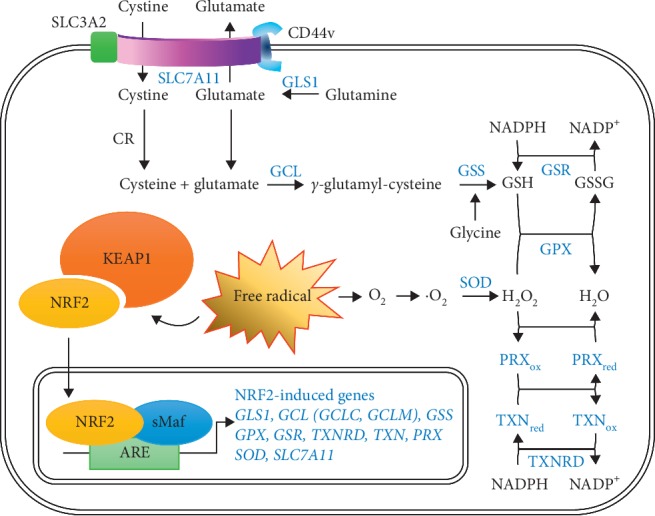
NRF2-mediated pathway of glutathione synthesis. The expression of antioxidant proteins (blue) can be induced by the Keap1-NRF2 pathway. Cystine introduced into the cytoplasm via xCT (SLC7A11) is converted to cysteine by cystine reductase (CR). Glutamine is converted to glutamate by glutaminase 1 (GLS1). Glutamate and cysteine bind and are transformed to *γ*-glutamyl-cysteine by glutamate-cysteine ligase (GCL). Glutathione synthetase (GSS) induces glutathione (GSH) synthesis via inducing a covalent bond between *γ*-glutamyl-cysteine and glycine. Glutathione peroxidase (GPX) oxidizes GSH to GSSG with H_2_O_2_ reduction to H_2_O. GSSG can then be reduced by glutathione reductase (GSR). In addition to the GSH system, the thioredoxin (TRX) system (thioredoxin reductase (TXNRD), thioredoxin (TXN), and peroxiredoxin (PRX)) also comprises NRF2-mediated antioxidant proteins.

NADH generated during glycolysis and the tricarboxylic acid (TCA) cycle transfers two electrons to complex I (NADH: ubiquinone oxidoreductase) in the mitochondrial matrix [50]. FADH_2_ produced in the TCA cycle also transfers electrons to complex II (succinate dehydrogenase) [51]. Electrons from complexes I and III are subsequently transferred to ubiquinone, complex III (coenzyme Q cytochrome C reductase), and complex IV (cytochrome c oxidase) [52]. Complex IV transfers electrons to oxygen, and then, reduced oxygen forms water via a reaction with two hydrogen ions [53]. H^+^ ions are pumped into mitochondrial intermembrane spaces through complexes I, III, and IV during electron transfer processes, and a hydrogen ion gradient is generated around the inner mitochondrial membrane [54]. ATPs are synthesized using the energy induced by H^+^ ions entering into the intermembrane spaces through complex V (ATP synthase) [55]. Electrons leaked from complexes I and III partially reduce oxygen to form ROS, such as superoxide during OXPHOS [56]. Approximately 90% of intracellular ROS are estimated to be produced during OXPHOS in mitochondria [57]. Proper ROS levels stimulate cell proliferation, mediate signal cascades, and initiate immune responses, but excessive ROS levels lead to cell death [58]. Therefore, when ROS are induced, the cell activates antioxidant mechanisms for homeostasis [59]. Once superoxide is produced in mitochondria, manganese superoxide dismutase (MnSOD/SOD2) converts it to hydrogen peroxide [60]. Hydrogen peroxide, one of the ROS, must be converted to water. Glutathione (GSH) and thioredoxin (TRX) systems controlled by the Kelch-like ECH-associated protein 1-nuclear factor erythroid 2-related factor 2 (KEAP1-NRF2) pathway are required for this chemical change (Figure 2) [61]. Intracellular oxidative stress can directly and immediately promote the expression of antioxidant enzymes via the KEAP1-NRF2 pathway [62]. KEAP1 induces NRF2 degradation through its polyubiquitination on the NRF2-ECH homology-like domain 2 (Neh2) domain of NRF2 in cells under normal conditions [63]. However, when intracellular ROS are induced, ROS oxidize cysteine residues in the intervening region (IVR) of KEAP1, and structurally modified KEAP1 is detached from NRF2 [64]. Free NRF2 translocated into the nucleus forms a heterodimer with small musculoaponeurotic fibrosarcoma (sMaf), and the transcriptional factors then bind to antioxidant response elements (AREs) to promote antioxidant gene expression [65]. SLC7A11 (xCT) forms a complex known as the xCT system, with CD98 (SLC3A2 or 4F2) and the CD44 variant isoform (CD44v) [66]. CD98 and CD44v contribute to the stability of the xCT system; in particular, CD44v helps to locate xCT on the cell surface [67]. The xCT system, a cystine-glutamate antiporter, mediates the influx of extracellular cystine and the efflux of glutamate transformed by glutaminase 1 (GLS1) from glutamine [68]. One cystine molecule transported into the cell is reduced to two cysteine molecules by cystine reductase (CR) [69]. Cysteine and glutamate are synthesized to *γ*-glutamyl-cysteine via heterodimeric glutamate-cysteine ligase (GCL) consisting of GCLC (catalytic subunit) and GCLM (modulating subunit), and the ligated product is then converted to glutathione via the glutathione synthetase- (GSS-) mediated addition of glycine [70]. Glutathione peroxidase (GPX) promotes H_2_O_2_ reduction to H_2_O during the oxidation of GSH (reduced form) to glutathione disulfide (GSSG, oxidized form) [71]. Oxidized GSSG is returned to the reduced form of GSH by glutathione reductase (GSR) [72]. In addition to the GSH system, NRF2 induces gene expression of the TRX system [73]. Thioredoxin reductase (TXNRD) oxidizes NADPH to NADP^+^ to reduce the oxidized form of thioredoxin (TXN) [74]. The reduced TXN induces H_2_O_2_ reduction to H_2_O through the redox reaction of peroxiredoxin (PRX) [75]. Thus, the KEAP1-NRF2 pathway can be regarded as an essential mechanism for antioxidant defense.

### 2.2. mTOR-Dependent Antioxidant Mechanism in Solid Tumors

**Figure 3:**
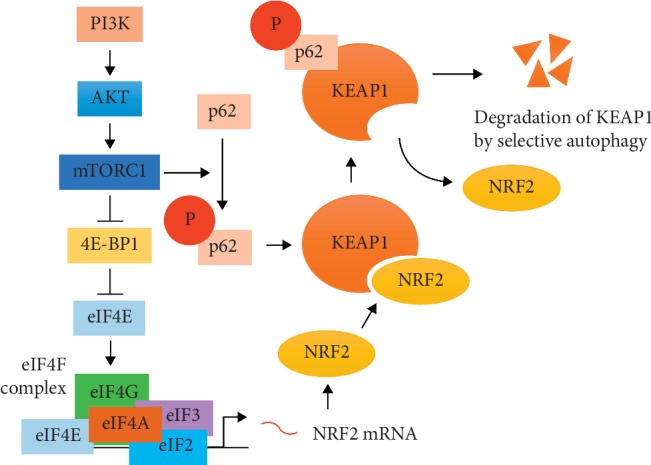
The effect of mTORC1 on NRF2 expression and activation. The activation of mTORC1 promotes the transcriptional activation of eIF4F. The eIF4F complex can induce NRF2 mRNA expression. NRF2 translocation into the nucleus is restricted because of its attachment to KEAP1. However, mTORC1 activation can induce the phosphorylation of p62 (at serine 351) and promote NRF2 translocation into the nucleus through p62-mediated KEAP1 degradation.

ROS can be controlled by NRF2-mediated antioxidant mechanisms in normal cells, consequently maintaining cellular homeostasis [76]. However, in various solid tumors, because antioxidant mechanisms are activated at higher levels than in normal cells, tumor cells can be more tolerant to excessive ROS levels than normal cells [77]. NRF2-dependent antioxidant enzymes such as SOD, glutathione peroxidase (GPX), glutathione reductase (GSR), peroxiredoxin (PRX), and thioredoxin reductase (TXNRD) are upregulated in tumor cells, and high expression of these proteins is associated with poor prognosis in tumor patients (Table 1) [78–83]. Mitochondrial SOD2 was shown to be highly expressed in ovarian cancer patients and contribute to antitumor therapy resistance [84]. Non-small-cell lung cancer (NSCLC) cells overexpressing GPX1 were resistant to cisplatin-induced ROS through PI3K/AKT pathway activation [85]. In pancreatic cancer cells, GPX4 was shown to be essential for the maintenance of stemness, and PRX1 was required for p38-mediated invasion [86]. A high level of PRX2 in colorectal cancer patients was associated with tumor progression and poor diagnosis [87]. Inhibition of GSR and TXNRD attenuated tumor growth in an NSCLC patient-derived xenograft model [88]. High *γ*-glutamylcysteine synthetase (*γ*-GCS) activity in human HCC cells was related to radioresistance [89]. In general, radiation promotes GSH synthesis [90]. Because rapamycin and everolimus can affect GSH synthesis, the use of mTOR inhibitors might increase the effectiveness of radiotherapy [91, 92]. In addition to antioxidant enzymes, the NRF2-dependent xCT system could promote the radioresistance of solid tumors. xCT was related to radioresistance in human breast cancer and mouse melanoma cell lines [93, 94]. CD98 contributed to radioresistance in head and neck squamous cell carcinoma [95]. CD44v induced radioresistance in human pancreatic cancer cells via increasing xCT stability [96]. These results suggest that antioxidant proteins controlled by the KEAP1-NRF2 pathway could be factors that make tumor therapy difficult. Therefore, targeting the KEAP1-NRF2 pathway might be a strategy to improve the efficacy of radiotherapy. In a recent study, xCT was reported to be modulated by mTOR signaling in human melanoma subjected to radiation [97]. The transcriptional activity of NRF2 can be regulated by mTOR inhibitors. Temsirolimus suppressed NRF2 translocation into the nucleus in RCC4 cells, a human renal cell carcinoma (RCC) cell line [98]. Everolimus reduced the phosphorylation of NRF2 in ARPE-19 adult retinal pigment epithelial cells [99]. In general, KEAP1 is important for the transcriptional regulation of NRF2. Intracellular ROS promote NRF2 activity through the structural transformation of KEAP1 [64]. KEAP1 can also be dissociated from NRF2 by mTORC1. mTORC1 phosphorylates serine 351 in the Keap1‐interacting region (KIR) of sequestosome 1 (SQSTM1/p62) [100]. Phosphorylated SQSTM1/p62 promotes KEAP1 degradation during selective autophagy [101]. Degradation of KEAP1 via autophagy activation does not necessarily occur in an mTOR-dependent manner [102, 103]. Moreover, mRNA expression of NRF2 is dependent on the transcriptional activity of eIF4F [104]. Activated mTORC1 signaling disturbs the inhibitory effect of 4E-BP1 on eIF4E through the phosphorylation of 4E-BP1 [105]. In other words, mTORC1 signaling activation could mediate NRF2 expression. Tuberous sclerosis complexes 1 and 2 (TSC1 and TSC2), also known as hamartin and tuberin, respectively, attenuate mTORC1 activity via inhibiting Ras homolog enriched in brain (Rheb) [106]. In a TSC1-null bladder cancer xenograft model, not only mTORC1 hyperactivation but also upregulated NRF2, GCLM, GCLC, and GSR expressions were observed [107]. Overall, previous studies suggest that the antioxidant mechanism could be mediated by NRF2 and that NRF2 expression and activation could be dependent on mTOR signaling (Figure 3).

### 2.3. The Regulation of Antioxidant Defense via mTOR Inhibition in Solid Tumors Subjected to Radiation

**Figure 4:**
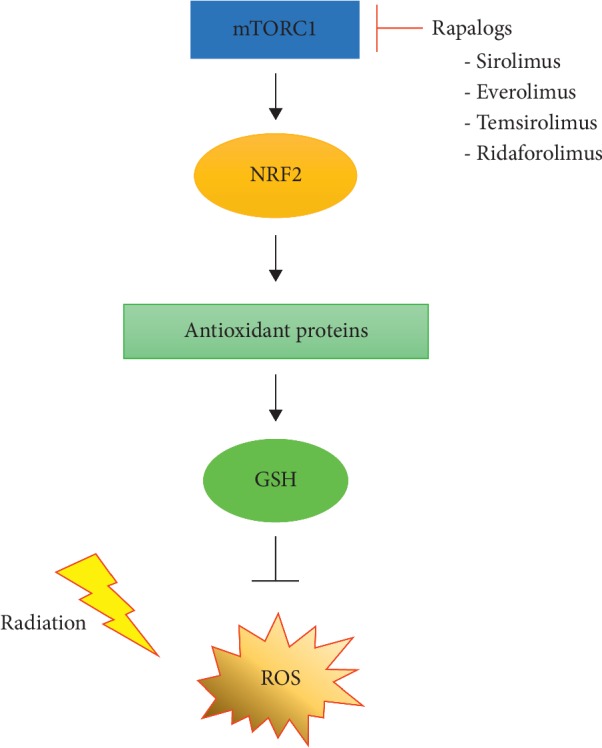
A strategic proposal for enhancing tumor sensitivity to radiation via inhibiting mTOR signaling. Activated mTORC1 signaling in tumors might effectively eliminate radiation-induced ROS through promoting antioxidant mechanisms. However, inhibiting mTOR signaling might attenuate the antioxidant KEAP1-NRF2 pathway. Therefore, blockade of mTOR signaling in tumors might be an alternative strategy to increase tumor susceptibility to radiation.

Tumor tissues are formed by the uncontrolled proliferation of cancerous cells [122]. As tumor tissues develop, it is difficult for some tumor cells to obtain nutrients and oxygen because blood vessels are unevenly distributed in tissues [123]. In particular, OXPHOS and ROS production in mitochondria depend on the supply of oxygen [124]. Therefore, the oxygen levels in the tumor microenvironment should be considered in the regulation of antioxidant defense through mTOR inhibition in solid tumors. Mitochondrial OXPHOS is stably induced in tumor cells under sufficient oxygen supply, but not under pseudohypoxic conditions [125]. Although higher ROS levels are induced in tumor cells than in normal cells, ROS levels could be properly controlled through the KEAP1-NRF2 pathway [126]. Because tumor cells are exposed to nutrients and various growth factors, such as epidermal growth factor (EGF), fibroblast growth factor (FGF), and vascular endothelial growth factor (VEGF), mTOR signaling activation is promoted in the cells under normoxic conditions [127]. Moreover, radiation promotes not only ROS generation but also OXPHOS. In general, radiation causes mitochondrial damage and ROS production [128]. Irradiated tumor cells might use metabolism through glycolysis rather than OXPHOS because of mitochondrial damage [129]. However, irradiation with a single dose of 5 Gy promoted OXPHOS via mTOR-mediated enzymatic inhibition of hexokinase II in tumor cell lines (breast cancer MCF-7 cells, colon cancer HCT116 cells, and glioblastoma U87 cells) associated with the Warburg effect [130]. This implies that radiation might enhance OXPHOS in rapidly proliferating tumor cells. Thus, the inhibition of mTOR signaling might make tumor cells sensitive to radiation-induced ROS via the attenuation of NRF2-mediated antioxidant mechanisms under normoxic conditions. Unlike normal oxygen conditions, hypoxia can be caused in tumor cells located in tumor tissues with little blood vessel distribution [131]. The levels of OXPHOS and ROS generation could be reduced in tumor cells under persistent hypoxic conditions [132]. Hypoxia promotes the stability of hypoxia-inducible factor-1*α* (HIF-1*α*) [133]. HIF-1*α* induces regulated in development and DNA damage response 1 (REDD1) expression [134]. mTOR activity is completely inhibited in REDD1-overexpressing cells under normoxic conditions, and continuous hypoxia can weaken its activity in tumor cells [135, 136]. Therefore, it might be difficult to expect the regulatory effects of mTOR inhibition on tumor antioxidant defense under extreme hypoxic conditions. However, under these conditions, not only might all mitochondrial OXPHOS, ROS generation, and mTOR activity be reduced but also necrotic cell death could be induced in hypoxic tumor tissues [137]. Therefore, the inhibition of mTOR signaling is expected to make tumor cells sensitive to radiation-induced ROS via KEAP1-NRF2 pathway attenuation, except for in the continuously hypoxic regions in the tumor tissues (Figure 4).

## 3. Cell Death Mechanisms Induced by mTOR Inhibition during Radiotherapy

### 3.1. The Promotion of Cell Death by mTOR Inhibition in Irradiated Tumors

Radiation induces reductions in tumor volumes and inhibits tumor metastasis [138, 139]. mTOR inhibitors, such as rapalogs, have been demonstrated to increase the radiosensitivity of tumors in previous studies. Rapamycin is an efficient drug to enhance radiosensitivity in various tumor types [39, 140]. Everolimus suppressed epithelial-mesenchymal transition (EMT) and angiogenesis [35, 40]. Everolimus also inhibited tumor stemness and recurrence [141, 142]. Therefore, the use of mTOR blockers might be a rational approach when treating tumor patients using radiotherapy. Ionizing radiation mainly causes apoptosis in tumor cells [143]. Various types of cell death including necrosis, apoptosis, pyroptosis, ferroptosis, and autophagy could be induced depending on the properties of adjuvants [144–147]. The inhibition of mTOR signaling induces autophagy [148]. Tumor cells can survive via autophagy under nutrient-depleted conditions, but autophagy can cause cell death in tumors depending on the surrounding environment [149]. Resistance to apoptosis and radiation has been observed in various tumor types [8]. Thus, targeting mTOR signaling can be effective in inducing autophagic cell death in apoptosis-resistant tumors during radiotherapy [150].

### 3.2. mTOR Inhibition and Ferroptosis in Radiotherapy

Induction of cell death in tumors is an important strategy to promote tumor radiosensitivity. The induction of ferroptosis has recently been reported to improve antitumor therapy in a tumor study [151]. Ferroptosis is a type of cell death with morphology similar to necrosis, accompanied by excessive iron accumulation and lipid peroxidation [152]. However, unlike necrosis, ferroptosis is a form of regulated cell death mediated by the Fenton reaction [153]. A recent study showed that IFN-*γ* secreted by CD8^+^ T cells can induce ferroptosis in tumor cells during immunotherapy with anti-CTLA-4 and anti-PD1 [154]. However, further investigations are needed to determine whether ferroptosis can be induced in tumors to improve radiosensitivity during tumor radiotherapy. The inhibition of mTOR activation can induce autophagy in tumor tissues. Autophagy promotes the degradation of ferritin, which contains Fe^2+^, thereby increasing the Fe^2+^ level in the cytoplasm [155]. mTOR overexpression suppressed ferroptosis, whereas mTOR depletion enhanced ferroptosis in cardiomyocytes [156]. High levels of NRF2 and GSH inhibited ferroptosis, but mTOR inhibition reduced the inhibitory effects of both NRF2 and GSH on ferroptosis [157]. This indicates that ferroptosis might be associated with the autophagic process [158]. In a recent study, nanoparticles bound to erastin and rapamycin efficiently suppressed tumor growth [159]. Therefore, the inhibition of mTOR signaling might induce ferroptosis as well as autophagy in irradiated solid tumors.

## 4. Conclusion

Ionizing irradiation should not only reduce tumor volume but also suppress tumor recurrence in tumor radiotherapy. However, because radioresistance is observed in various solid tumors, the use of proper adjuvants is important to effectively treat tumor patients. Adjuvants used in radiotherapy should not only increase the sensitivity of tumors to radiation but also induce optimal cell death. In particular, the induction of regulated cell death (RCD) by adjuvants is imperative in host immunity during radiotherapy. In this review, the effects of mTOR inhibition on tumor radiosensitivity were discussed. Tumor cells can be resistant to ROS through mTOR-mediated antioxidant defense. Thus, the inhibition of mTOR signaling in these tumor types could attenuate the expression of antioxidant enzymes and make tumors sensitive to ROS. In addition, the inhibition of mTOR might induce RCD, such as autophagy and ferroptosis, in tumors during irradiation. There is a lack of validation of the regulatory effects of mTOR inhibition on antioxidant mechanisms during radiotherapy. Thus, further studies on mTOR inhibitors are required for efficient radiotherapy. In this review, the attenuation of the KEAP1-NRF2 pathway through inhibiting mTOR signaling has been suggested as an approach to enhance radiosensitivity.

## Figures and Tables

**Table 1:** Glutathione synthesis-related proteins in the poor prognosis of tumor patients.

	Type of cancer	Level	Effects	Ref.
Glutathione peroxidase (GPX)	Breast cancer	High	Metastasis↑	[80]
			Patient mortality↑	
	Bladder cancer	High	Recurrence↑	[91]
	Oral squamous cell carcinoma	High	Metastasis↑	[92]
			Poor survival↑	
	Gastric carcinoma	High	Metastasis↑	[108]
			Progression↑	
	Hepatocellular carcinoma	High	Poor prognosis↑	[109]
			Recurrence↑	

Glutathione reductase (GR)	Glioblastoma	High	Drug resistance↑	[81]
			Poor survival↑	

Glutamate-cysteine ligase (GLC)	Hepatocellular carcinoma	High	Progression↑	[110]
			Drug resistance↑	
			Poor survival↑	
	Breast cancer	High	Drug resistance↑	[111]
	Melanoma	High	Malignancy↑	[112]
	Lung adenocarcinoma	High	Recurrence↑	[113]
			Poor survival↑	

Glutaminase 1 (GLS1)	Colorectal cancer	High	Metastasis↑	[114]
			Poor prognosis↑	
			Poor survival↑	
	Hepatocellular carcinoma	High	Malignancy↑	[115]
			Poor prognosis↑	
			Cancer stem cells	
	Breast cancer	High	In HER2-type↑	[116]

SLC7A11 (xCT)	Colorectal cancer	High	Recurrence↑	[117]
			Poor survival↑	
	Non-small-cell lung carcinoma	High	Poor survival↑	[118]
	Glioma	High	Poor survival↑	[119]
	Hepatocellular carcinoma	High	Poor survival↑	[120]
	Breast cancer	High	Poor prognosis↑	[121]
